# Scatter-plate microscope for lensless microscopy with diffraction limited resolution

**DOI:** 10.1038/s41598-017-10767-3

**Published:** 2017-09-06

**Authors:** Alok Kumar Singh, Giancarlo Pedrini, Mitsuo Takeda, Wolfgang Osten

**Affiliations:** 1 Institut für Technische Optik and Stuttgart Research Center of Photonic Engineering (SCoPE), University of Stuttgart, Pfaffenwaldring 9, 70569 Stuttgart, Germany; 2 Center for Optical Research and Education (CORE), Utsunomiya University, Yoto 7–1–2, Utsunomiya, Tochigi, 321–8585 Japan

## Abstract

Scattering media have always been looked upon as an obstacle in imaging. Various methods, ranging from holography to phase compensation as well as to correlation techniques, have been proposed to cope with this obstacle. We, on the other hand, have a different understanding about the role of the diffusing media. In this paper we propose and demonstrate a *‘scatter-plate microscope’* that utilizes the diffusing property of the random medium for imaging micro structures with diffraction-limited resolution. The ubiquitous property of the speckle patterns permits to exploit the scattering medium as an ultra-thin lensless microscope objective with a variable focal length and a large working distance. The method provides a light, flexible and cost effective imaging device as an alternative to conventional microscope objectives. In principle, the technique is also applicable to lensless imaging in UV and X-ray microscopy. Experiments were performed with visible light to demonstrate the microscopic imaging of USAF resolution test target and a biological sample with varying numerical aperture (NA) and magnifications.

## Introduction

Traditional lens-based microscope objectives have fixed focal length, short working distance, limited depth of focus and magnification, which often sets restrictions in applications. The correction of aberrations for microscope objectives requires a combination of spherical lenses (sometimes more than 10), which increases complexity, bulkiness, and price. Aspherical elements can reduce the complex multiple-lens system and make the objectives more compact, but they demand more complex fabrication and testing procedures, which also raises the cost. Hybrid systems, integrating diffractive and refractive elements, have been developed, but they still need solutions for enhanced diffraction efficiency and stray light reduction. Techniques have been proposed to replace conventional lens systems for microscopy purposes. Digital holographic microscopy^1–3^ phase retrieval techniques^4–6^ and ptychography^7^ are few such techniques which are successfully demonstrated. However, holography suffers from coherent noise, and phase retrieval and ptychography require sequential recording of multiple diffraction patterns followed by time consuming signal processing.

Imaging through the scattering media has potentially wide range of applications. Freund proposed in 1988 an intensity-correlation-based technique, which permits lensless imaging of an object through a scattering medium by making use of the memory effect^8, 9^. This principle was further exploited by combining it with phase retrieval algorithms^10, 11^ for diffraction limited imaging. A color imaging scheme was also presented^12^. Many other methods have been developed in recent times for this purpose, among which are coherence and time gating^12–17^ phase compensation^18–26^ and unconventional holography^27–32^. However, microscopic imaging through diffusing media has been performed exclusively with traditional lens-based microscope objectives being combined with the above mentioned methods^23, 25, 33–36^. In these approaches, the diffusing media play no constructive role but have always been treated negatively as a nuisance, except for a few instances where diffusing media have been utilized successfully for resolution enhancement in microscopy^37, 38^ and speckle scanning endoscopy^39, 40^.

Whereas Katz *et al*.^10^ and Vellekoop *et al*.^19^ gave important contributions to the development of imaging that can cope with a scattering medium, we take an alternative approach that does not regard the scattering medium as an obstacle but exploits its lens-like function^8, 41^, for the imaging of objects. Specifically, we propose a new imaging device called ‘*scatter-plate microscope’* in which a conventional objective lens is replaced by a simple scatter plate. The scatter-plate microscope has the following unique characteristics that are not available with traditional microscopes:

- extremely thin and light (suitable for low cost production),
- variable focal length and magnification (self-adaptive to any conjugate image planes),
- variable NA and field of view,
- flexible working distances extendable as desired,
- compatible in reflection and transmission modes,
- immune to phase disturbances and aberrations,
- easy to fabricate with scalability in device size, robust to environmental changes.

Thus, a single low-cost scattering plate can perform the tasks of various microscope objectives with different magnifications, NAs and focal lengths. A relatively wider field of view (FOV) and variable depth of focus (DOF) are exclusive features of the presented technique. Whereas autocorrelation techniques, combined with phase retrieval algorithm, are restricted to the imaging of sparse objects only^10^, our cross-correlation technique, combined with a reference point source, is capable of imaging complex objects. To our knowledge, this is the first experimental demonstration of lensless microscopy using a scatter plate.

It also opens up the possibility of utilizing the scattering media as imaging lenses for x-ray and ultraviolet wavelength. For x-rays, the scattering properties have been extensively studied^42^, so that an appropriate diffusing layer can be chosen.

Thus we present a technique that can turn a costless scattering surface into a high resolution microscope objective. We demonstrate by experiments, the high potential of the scatter-plate objectives for microscopic imaging. Experiments were performed on USAF test target and biological samples to verify our claims.

## Materials and Methods

### Principle

**Figure 1:**
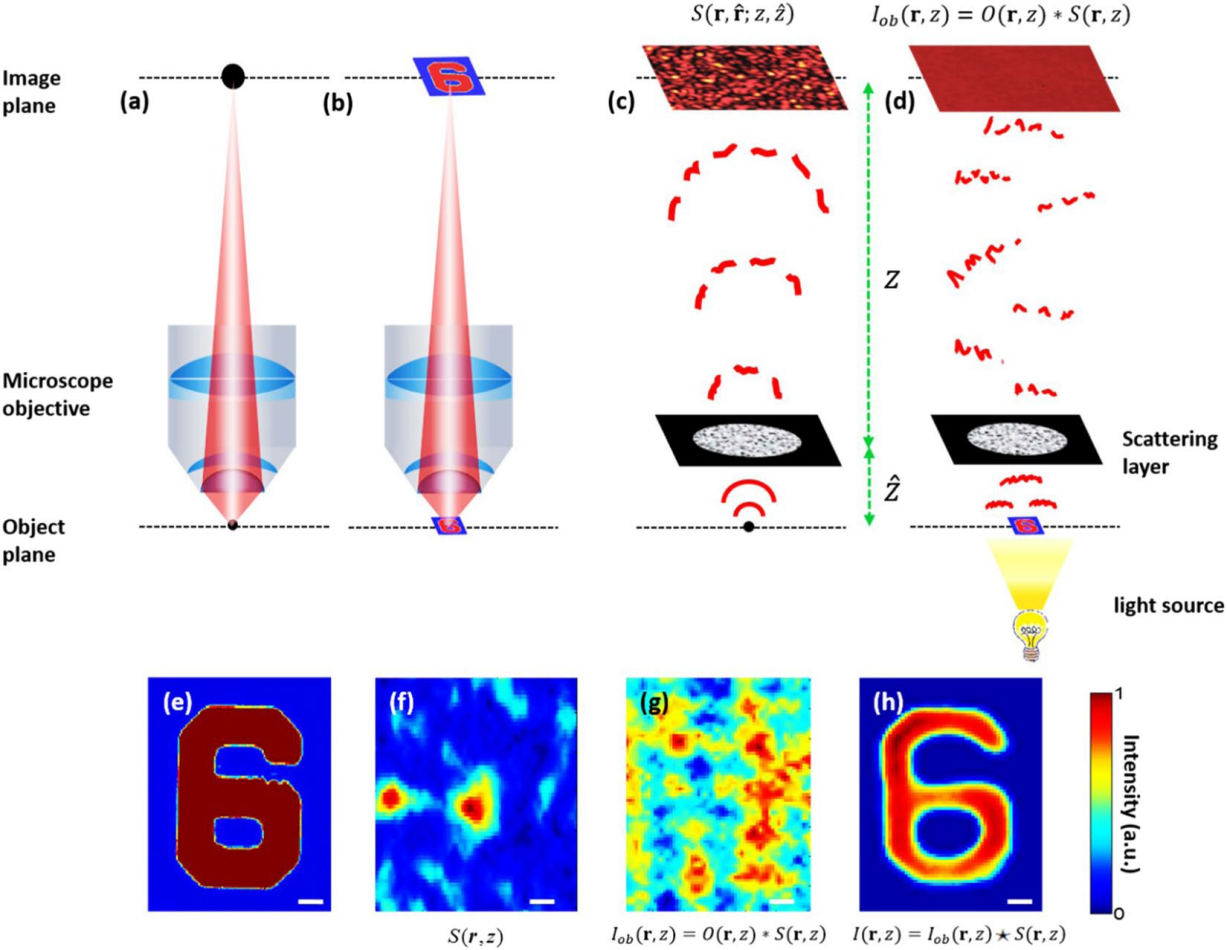
Microscope imaging systems: (**a**) PSF of a conventional microscope objective (**b**) conventional microscope imaging (**c**) PSF of a scattering layer, which in this case is a speckle pattern $S({\boldsymbol{r}},\hat{{\boldsymbol{r}}},z,\hat{z})$, (**d**) microscope imaging using a thin scattering layer (‘scatter-plate microscope’). The object is placed close to the scattering layer at $\hat{z}$, and the aperture size is increased to achieve a high numerical aperture. The image plane is far at a distance $z$ from the scattering layer, to have a larger magnification. The images are produced from the cross-correlation of the object intensity distribution ${I}_{ob}({\boldsymbol{r}},z)$ (in the image plane) and the PSF $S({\boldsymbol{r}},\hat{{\boldsymbol{r}}},z,\hat{z})$. (**e**) Conventional microscope image of numeral 6, (**f**) a part of the PSF of the scattering layer (speckle pattern), (**g**) a part of the recorded object intensity distribution and (**h**) the image reconstruction via intensity cross-correlation. The scale bar is 3 *µm* in object space.

Like other imaging systems, a microscope objective can be characterized by its point spread function (PSF), which is the output intensity distribution in the image plane when a point source is placed in the input (object) plane. The objective produces a magnified image of the object. On the assumption of shift invariance and incoherent illumination, the image is given by the convolution of the object intensity distribution with the PSF^43^. Figure 1(a) and (b) show the impulse response of a conventional microscope objective and the imaging operation, respectively. A scatter-plate-microscope system can be defined in the same way, except for the fact that the PSF in this case is a speckle pattern $S({\boldsymbol{r}},\hat{{\boldsymbol{r}}},z,\hat{z})$ as shown in Fig. 1(c).

$\hat{z}$ and $z$ are the distances of the object plane (point source in this case) and the image plane from the scattering layer, whereas $\hat{{\boldsymbol{r}}}$ and ${\boldsymbol{r}}$ are the lateral position vectors in the respective planes.

For a thin scattering layer, this PSF remains shift invariant in the vicinity of the point source i.e. $S({\boldsymbol{r}}+{\rm{\Delta }}{\boldsymbol{r}},\hat{{\boldsymbol{r}}}+{\rm{\Delta }}\hat{{\boldsymbol{r}}},z,\hat{z})=S({\boldsymbol{r}},\hat{{\boldsymbol{r}}},z,\hat{z})$, where ${\rm{\Delta }}\hat{{\boldsymbol{r}}}$ and ${\rm{\Delta }}{\boldsymbol{r}}=-\,(z/\hat{z}){\rm{\Delta }}\hat{{\boldsymbol{r}}}$ are lateral shifts in the object and the image planes, respectively. This phenomenon is called memory effect^9^, which states that each elementary point source that constitutes the object, placed in the vicinity of the reference point source, produces a shifted but similar speckle pattern to that of the point source. To attain a wide field of view, we enhance the memory effect by choosing a scatter-plate with a thin scattering layer. Thus if the PSF of the diffusing medium, placed at distance $\hat{z}$ from the point source, is known as $S({\boldsymbol{r}},\hat{{\boldsymbol{r}}},z,\hat{z})$, the object placed near the point source can be reconstructed from the cross-correlation between its intensity distribution ${I}_{ob}({\boldsymbol{r}},z)=O({\boldsymbol{r}},z)\ast S({\boldsymbol{r}},z)$ in the image plane and the PSF, i.e.

1 $$I({\boldsymbol{r}},z)={I}_{ob}({\boldsymbol{r}},z)\star S({\boldsymbol{r}},z)=[O({\boldsymbol{r}},z)\ast S({\boldsymbol{r}},z)]\star S({\boldsymbol{r}},z)$$

2 $$=O({\boldsymbol{r}},z)\ast [S({\boldsymbol{r}},z)\star S({\boldsymbol{r}},z)]$$

where ★ and * represent the cross-correlation and convolution operations, respectively. For simplicity, the auto-correlation of the PSF i.e. $[S({\boldsymbol{r}},z)\star S({\boldsymbol{r}},z)]$ can be assumed as a ‘$\delta$’ function with a constant background arising from the non-negativity of the PSF. Thus from equation (2) we can write the cross correlation of the PSF and the object speckle pattern as the convolution of the object intensity distribution and a *δ* function, which results in:

3 $$I({\boldsymbol{r}},z)=O({\boldsymbol{r}},z)+C.$$

Equation (3) gives the object reconstruction $O({\boldsymbol{r}},z)$, with an additive constant term *C*. In this way, the scatter- plate-based incoherent imaging system operates in three steps; (1) recording the PSF of the scattering layer, (2) recording the speckle intensity distribution of the object and (3) cross-correlating these two to reconstruct the object. This scheme was first proposed by Freund^8^ for imaging through a scattering medium. However, no experimental proof was presented, nor any applications for microscopy were suggested. Here we propose and experimentally prove the principle of a scatter-plate microscope, which reveals the great potential of a thin scattering layer as an imaging device in microscopy. An example of the imaging process is shown in Fig. 1. The numeral ‘6’, which is ~15 µm wide and ~20 µm tall, is imaged using a scatter-plate microscope. Figure 1(e) shows the conventional microscope image of the object, Fig. 1(f) and (g) show a part of the PSF of the scattering layer and the object intensity distribution in the imaging plane. Their cross correlation reconstructs the object image as shown in (h).

The necessary condition for such imaging is that the object and image plane should satisfy the lens equation $1/z+1/\hat{z}=1/f$, where $f$ is the focal length of the scattering lens. Whereas a conventional lens has a fixed focal length, the scattering lens has an adaptive focal length that adjusts itself automatically to satisfy this conjugate-image condition between the arbitrarily chosen planes at $z$ and $\hat{z}$. In analogy to a conventional lens, the lateral magnification of the scatter-plate - based imaging system can be defined as ${\rm{\Delta }}{\boldsymbol{r}}=-m{\rm{\Delta }}\hat{{\boldsymbol{r}}}$, where $m=-(z/\hat{z})$. Note that this magnification does not depend on the wavelength even though the speckle pattern changes with the wavelength. Thus the magnification of the system can be increased by increasing the distance of the image plane $z$ form the scatter-plate, or simply by moving the scattering lens toward the object. In a similar fashion, if the diameter of the input aperture $D$ is known, the resolution of the system is given by ${R}_{res}=0.61\,(\lambda /NA)\approx 1.22\lambda (\hat{z}/D)$, where $NA=n\,\sin \,\theta$ is the numerical aperture of the system. By bringing the object closer to the scatterer or by increasing the diameter of the aperture, the NA as well as resolution of the system can be increased. If the magnification is chosen such that $m\gg 1$, the scatter-plate plays the role of a microscope objective. The ease of fabricating a scatter-plate with a large aperture permits high NA imaging with a long working distance, which has been difficult with conventional objective lenses. Because the resolution observed in the object space can be much higher than that in the sensor space, we can overrule the practical resolution that is limited in the sensor space by the pixel size and the sampling theorem.

The depth of focus (DOF) of such a system is defined by the axial correlation length of the speckles i.e. $\delta z=6.7\lambda {(z/D)}^{2}$ ^44, 45^. For point shoot imaging systems (pinhole camera system), the diameter of the aperture is very small, so the DOF is infinity. In our case, the aperture of the imaging system has a defined finite value and so does the DOF. The field of view (FOV) of this system is limited by the decorrelation of the speckles or the maximum range of the memory effect and is given by $FOV=(\lambda \hat{z}/\pi t)$, where $t$ is the thickness of the diffusing layer^8, 10^. Thus by varying the parameter $\hat{z}$ (working distance between object and the scatter-plate) the FOV, resolution and the magnification of the imaging system can be changed. Let ${f}_{res}=1/{R}_{res}$ be the highest spatial frequency resolvable with the scatter plate objective, we have ${f}_{res}\times FOV=[D/(1.22\lambda \hat{z})]\times [(\lambda \hat{z})/(\pi t)]=D/(1.22\pi t)={\rm{constant}}$. This shows the trade-off relation between the resolution and FOV; interestingly it is achromatic with no dependence on the wavelength.

### Experimental setup

Experiments were performed on a 1951 USAF test target, which has a maximum resolution of 4.38 *µm*, to verify the microscopy principle (see Supplementary Figure S1). The object was illuminated with a narrow band spatially incoherent light source. A Nd:YAG laser with 532 *nm* central wavelength was employed as the light source during the experiments. The beam was split into two parts, one was fed into a rotating ground glass to create spatially incoherent light source to illuminate the object. Another part of the laser beam was utilized as a point source to detect the PSF of the diffuser. The object was placed in the close vicinity of the point source, so that it remains in the memory effect range but both were placed in the same plane. A variable diameter aperture was placed in front of the scattering layer to adjust the speckle size and also the NA of the system. The CCD was placed behind the diffuser at an appropriate distance so that the speckles are well sampled on the pixels. Once the PSF was recorded, the object was placed in the reference plane, off-axis to the reference point, and an intensity distribution was recorded. The cross-correlation operation was then performed digitally using Intel core i5 processor. To record the conventional microscope images a setup was prepared with a Nikon objective with 0.16 NA and 6.3X magnification was utilized. The monochromatic illumination was created by feeding the laser light into a rotating diffuser that destroys spatial coherence as earlier, and for white light image the object was illuminated with a white light source.

### Post processing

The cross-correlation images also contain the image of the point source, which was utilized to calculate the PSF. We cropped it out of the image to obtain desired image only. Normalization of the contrast was also performed.

## Results and Discussion

### High resolution imaging

**Figure 2:**
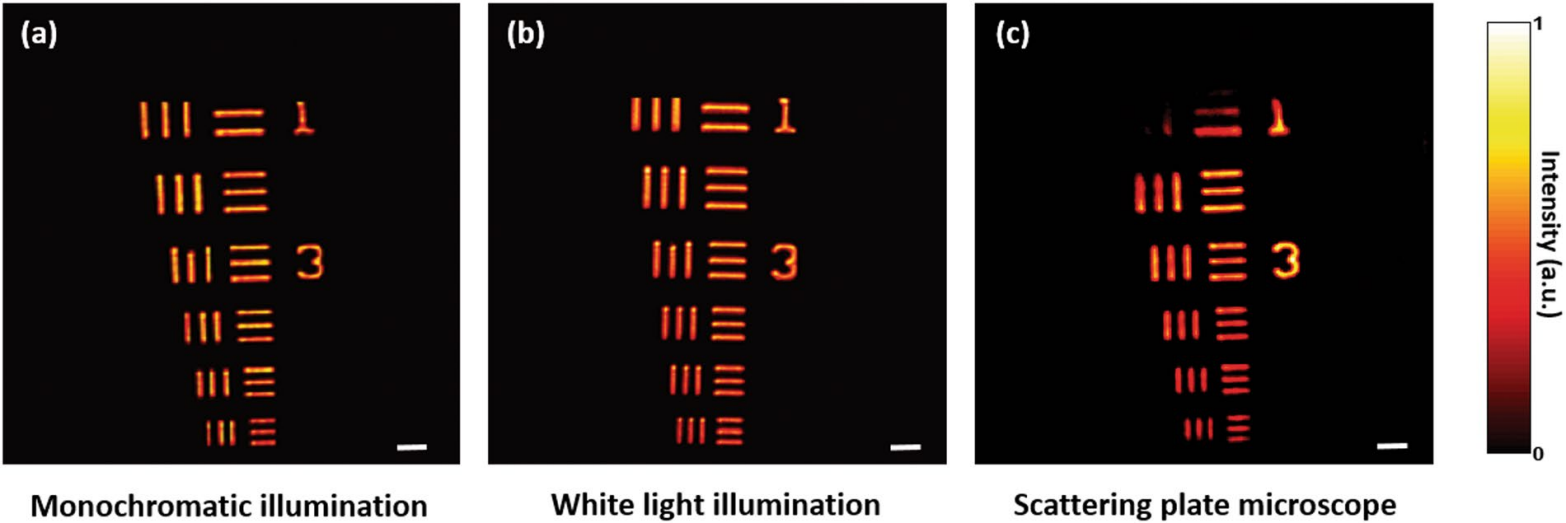
Comparison of conventional and scatter-plate microscope imaging: A 1951 USAF test target was used as an object. We imaged the highest resolution area of the test target, i.e. the 7th group, with smallest line thickness of 2.19 *µm*; **(a)** and (**b**) are the images obtained with a conventional microscope for NAs 0.16 under monochromatic and white light illuminations, respectively, whereas, (**c**) is their scatter-plate microscope counterpart for the same NA. The image quality, contrast and resolution of both techniques are almost equally good but the working distances of the scattering objective is comfortably long ($\hat{z}$ = 51 *mm*). The scale bar is 21.5 *µm*.

Our scatter-plate microscope is an incoherent imaging system that has no conventional lens elements. It images microscopic objects by making use of the non-localized speckle patterns generated by a scattering layer. Since $[S({\boldsymbol{r}},z)\star S({\boldsymbol{r}},z)]$ should approximate a *δ* function, a strong scatterer with a large scattering angle is a better choice for the objective, so that marginal rays can be scattered into the image sensor. For the experiments, we used poly-carbonate diffuser, which is a strong scatterer and is easily available in our laboratory. A 1951 United States Air Force (USAF) test target was used as the high resolution microscopic object. For each object position $\hat{z}$, the PSF of the optical system was recorded. The object was then illuminated with narrow-band spatially incoherent light with *λ* = 532 nm, created by passing the laser light through a rotating ground glass, and the intensity distribution of the scattered field was recorded with a CCD in the observation plane at $z$. The object was reconstructed by computing the cross-correlation between the PSF and the speckle intensity distribution generated by the object. Many experiments with different magnifications and numerical apertures using the same scattering lens were performed. A result of scatter-plate microscope imaging of a 1951 USAF test target and a comparison with images from a conventional microscope is shown in Fig. 2. The first two images shown in Fig. 2(a) and (b) were obtained with the conventional microscope under monochromatic and white light illuminations, respectively with NA = 0.16 objective, whereas the third image shown in Fig. 2(c) was captured using scatter-plate microscope while keeping the aperture diameter *D = *16 *mm* and the working distance (the object distance from the diffuser) $\hat{z}$ = 51 *mm*, resulting in the same numerical aperture NA = 0.16. In Fig. 2, all the elements of the 7th group were imaged and resolved. The width of 1 line of the 6th element in the group 7 is 2.19 *µm*. It can be seen that the quality of the images, in terms of resolution and contrast, of the scatter-plate microscope is almost comparable to that of a conventional microscope even though the edges look less sharp. Since the object reconstruction is based on the cross-correlation of two images, the quality of images of the scatter-plate microscope depends on the number of speckles available or number of contributing pixels in the averaging process. We used a SVS VISTEK CCD with 3280 × 4896 pixels, with unit pixel size 7.45 *µm* × 7.45 *µm*. Thus the total number of available correlation points (pixels) is over 16 million. The spatial averaging of speckle pattern on this large scale contributes to the good quality of reconstruction. The FOV of the scatter-plate microscope is limited by the memory effect of the scattering layer and is inversely proportional to its thickness, which implies that the FOV can be increased by using a thinner scattering layer.

**Figure 3:**
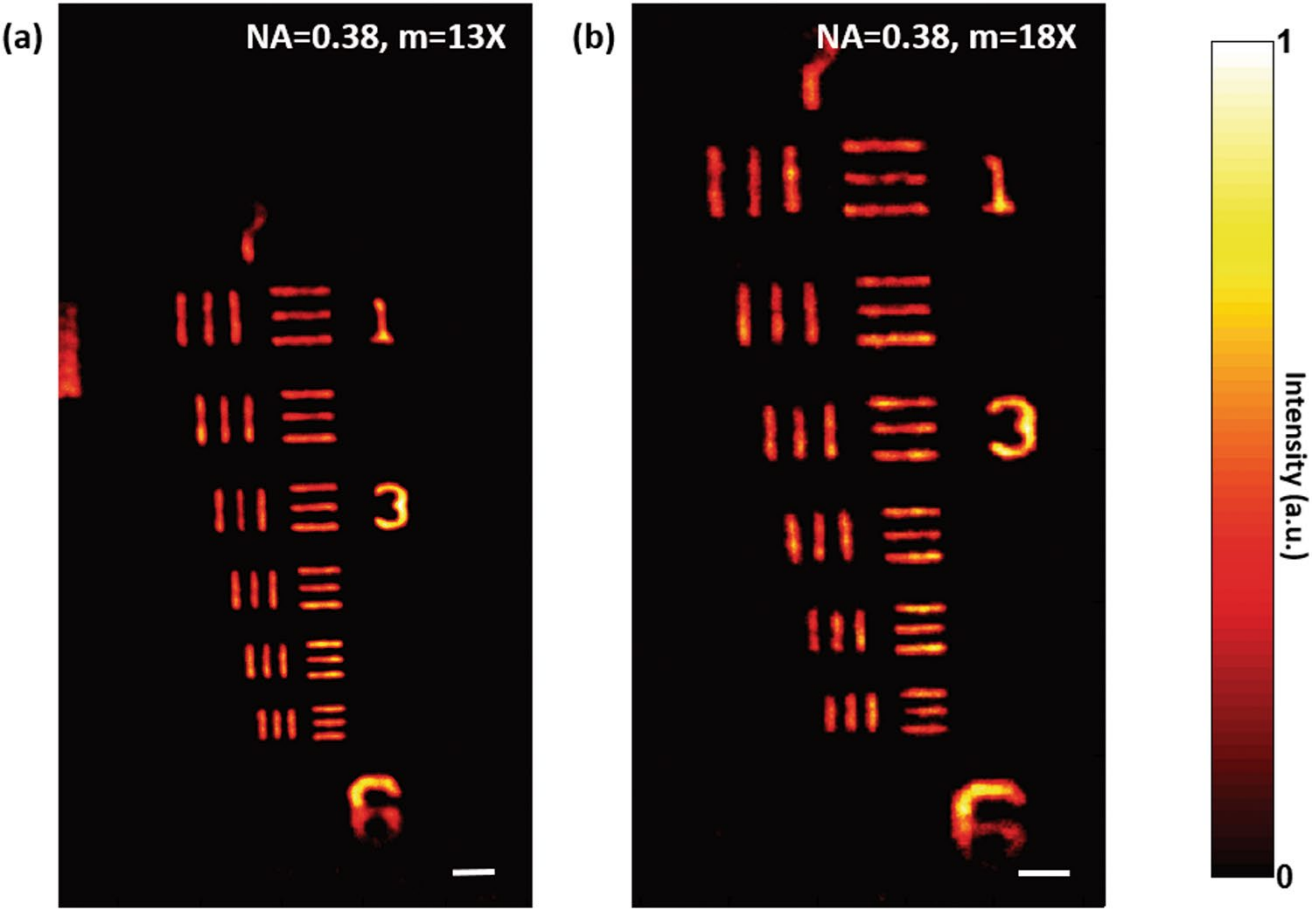
Imaging with varying magnification using a scatter plate. The 7th group of a 1951 USAF test target was imaged with NA = 0.38 and different magnifications. The Object was placed close to the diffuser at a distance $\hat{z}$ = 30.7 *mm* and the diameter of the aperture was *D = *25 *mm*. (**a**) *m = *13*X*, the CCD was placed at distances $z$ **= **400 *mm* from the diffuser. (**b**) *m = *18*X* and the CCD was shifted to $z$ **= **550 *mm*. The scale bar is 21.5 *µm*.

Next we performed the imaging with a fixed *NA* but with different magnifications. Figure 3(a) and (b) show the results of the scatter-plate microscope imaging with *NA* = 0.38 and the magnifications 13*X* and 18*X*, respectively. The numerical aperture was increased by increasing the aperture size to 25 *mm* and by bringing the diffuser close to the object. The object was placed at a distance 30.7 *mm* from the diffuser, and the CCD image sensor was positioned at different axial locations $z=-m\,\hat{z}$ depending on magnification *m*. Noticeable points here are that even at a relatively high numerical aperture, the working distance remains long and comfortable to work with, and also that imaging with variable magnification can be realized with the same diffuser while keeping the conjugate-image condition between the object and the CCD image senor. As pointed out above, by bringing the diffuser closer to the object and reducing the aperture size, the working distance, for the same NA, can be decreased if required.

### Imaging of biological samples

**Figure 4:**
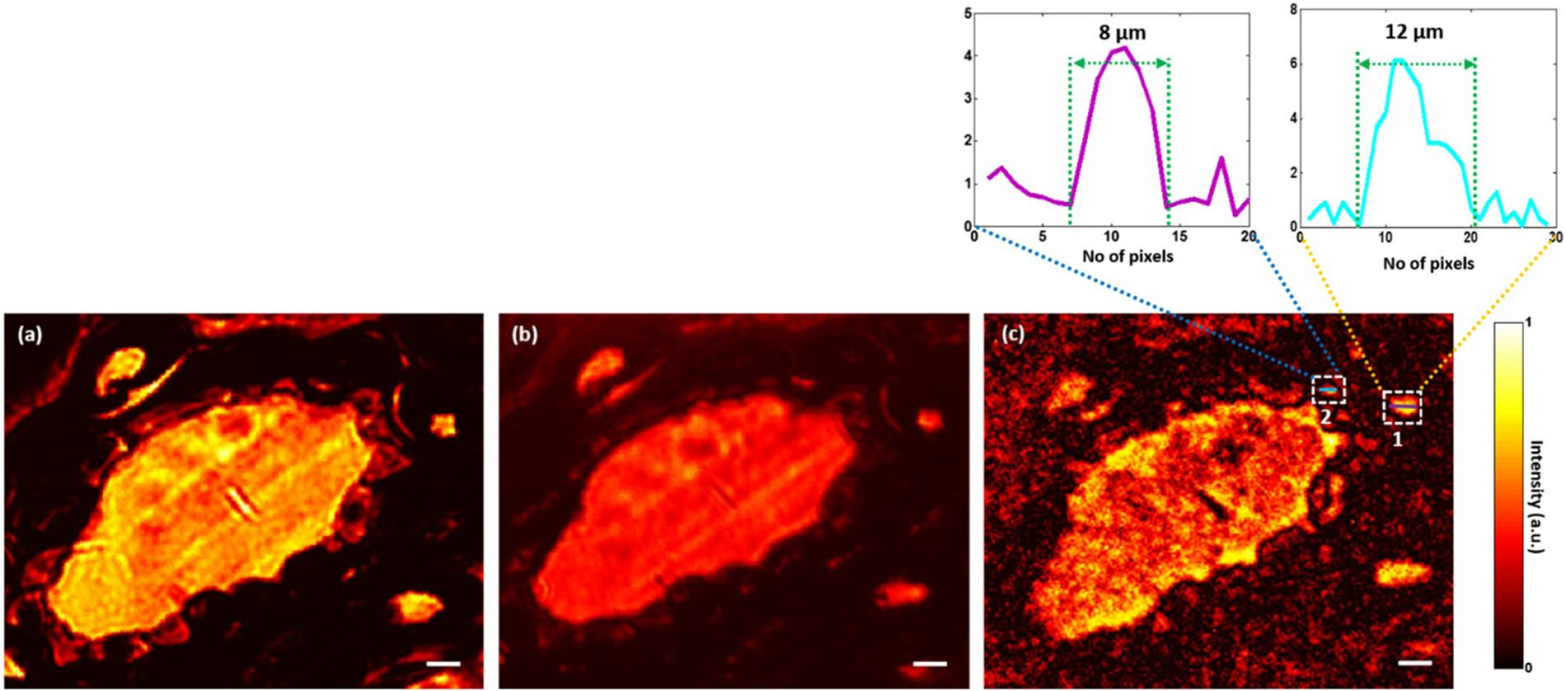
Microscopic imaging of a biological sample. A section of pine wood stem is imaged to show imaging performance of the scatter-plate microscope. (**a**) Conventional microscope image with monochromatic illumination and NA = 0.16. (**b**) Conventional microscope image with white light illumination and NA = 0.16. **(c)** Imaging performed using a scatter-plate microscope with NA = 0.16 and magnification 9X. The working distance in this case is 55 *mm*. Microscopic features of the tissue are resolved. Two microscopic structures of sizes 12 *µm* and 8 *µm* are encircled with dashed squares, and the cross-sectional plots are shown above in insets to verify the respective sizes. The scale bar is 20 *µm*.

A scatter-plate microscope, like the conventional microscope, can perform imaging of biological samples. Microscopic imaging was performed on a pine-wood stem, which has many miniature features, to show the imaging performance of the scatter-plate microscope on the biological samples. Figure 4 shows the comparison of the images between conventional microscope and scatter-plate microscope images with NA = 0.16. Figure 4(a) and (b) show the images of a part of the tissue obtained with a conventional microscope under monochromatic and white-light illuminations, respectively. The contrast of the image under the monochromatic illumination is better than with white light illumination and the structures are clearly visible.

As the microscope objective is color-corrected, the degradation of the image with white light may be attributed to the chromatic aberrations caused by the dispersion intrinsic to the miniature features of the pine-wood stem, where part of the object itself serves as a medium for light propagation.

The same feature was imaged using a scatter-plate microscope with spatially incoherent quasi-monochromatic light, and the image is shown in Fig. 4(c). Although the contrast of the image with the scatter-plate microscope does not surpass that of the conventional microscope under monochromatic illumination, in Fig. 4(a), it is better than the white light image shown in Fig. 4(b), and the structures are more obvious. The working distance in this case was $\hat{z}$ = 55 *mm* and the entrance pupil diameter was $D$ = 16 *mm*. As noted earlier the NA can be further increased by increasing the size of the entrance pupil or by reducing the working distance which can be adjusted on will. Two microscopic features of sizes 12 *µm* and 8 *µm* are encircled by dashed rectangles and their cross-sectional plot is shown above them in insets. The smaller features in the image are not clear in the conventional microscope image under white light illumination but are better resolved with scatter-plate microscope.

**Figure 5:**
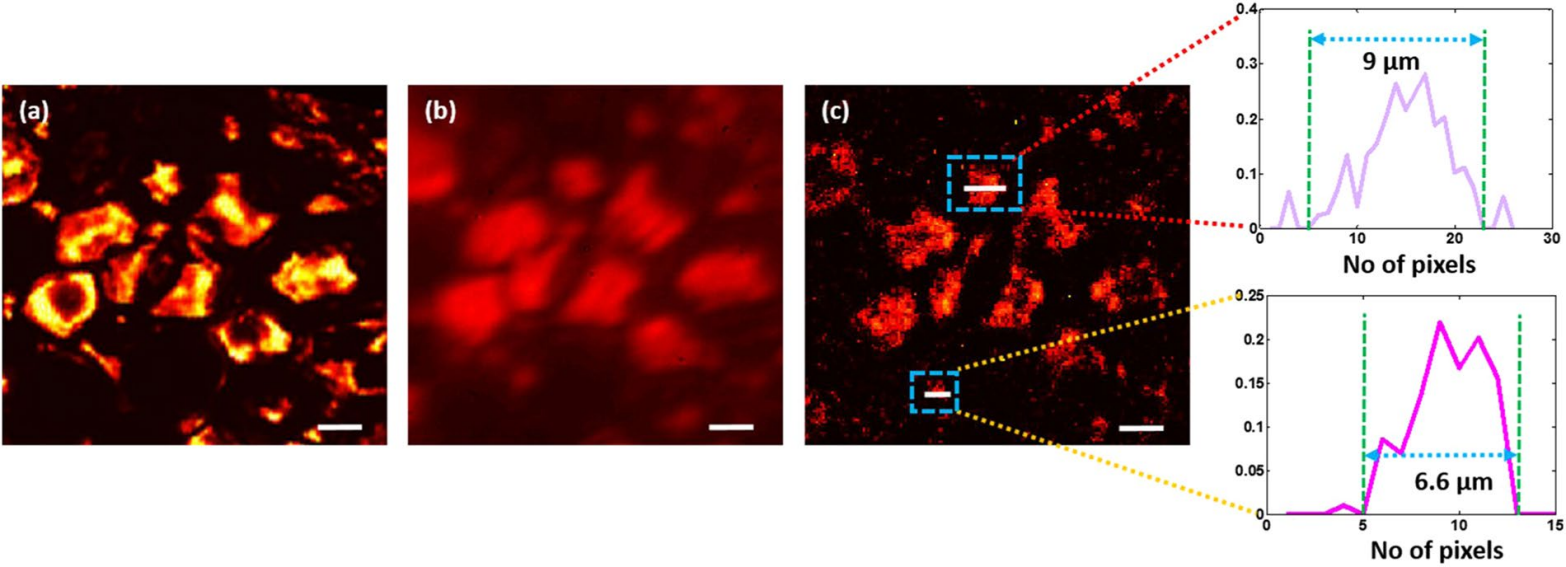
Imaging of a complex biological sample. The narrow band light source helps to reduce the noise considerably. At the same time, correlation based spatial averaging reduces the speckle noise in the reconstructed images. (**a**) and **(b)** are conventional microscope images of the central part of the pine-wood stem under monochromatic and white light illumination. White light image is marred by the blurs caused by chromatic aberrations and low contrast. (**c**) The scatter-plate microscope image with considerably less noise and high contrast. Two miniature features are encircled by dashed rectangles and their corresponding cross-sectional plots are shown in the inset on the right side. Note that the structures in Fig. 4 and this image are from different regions of the stem. The central region has discrete structures and is more absorbing. That is why the background light level is low here. For better presentation the signal levels have been normalized in this case. The scale bar is 10 *µm*.

Figure 5 shows another example of microscopic imaging of biological sample using the scatter-plate microscope. This time the central cross-section of the pine-wood stem, where thickness of the sample is comparatively higher and dispersion is more dominant, was imaged with conventional and scatter-plate microscopes. This section of the tissue includes many microscopic features of different shapes and sizes as shown in Fig. 5.

The numerical aperture of the imaging system is 0.16 and the FOV in this case is over 100 *µm*. Two microscopic features are encircled with dashed rectangles and the cross-sectional plots are shown on the right. The noise level in the reconstructed image is relatively low, which is evident from the cross-sectional plots.

## Conclusion

We proposed an unconventional scatter-plate microscope that utilizes a thin scattering layer as a microscope objective. Imaging operations were performed on various microscopic samples as the proof of the principle. The resolution, the contrast, and the quality of the images are nearly as good as conventional microscope images acquired with the same numerical aperture objectives. The scatter-plate microscope has the advantages of variable focal length, NA, magnification, flexible working distances and ability to work in reflection and transmission mode. Cost is another factor, the scatter-plate microscope objectives can be produced on large scale and on much lower prices than the current objectives. The presented microscope is capable of working with coherent and incoherent light sources. This way, any thin scattering media can be standardized for different working distances and can be used as a compact, lightweight and cost effective alternative to the existing bulky and expensive microscope objectives. In addition it may also be used in wide range of applications e.g. imaging lens in endoscopy etc. In principle, the presented technique should also be applicable in UV and x-ray regime as well, if the appropriate scattering surface is utilized.

The main restriction on the scatter-plate microscope is that the imaging process consists of two steps of speckle recording, first with a reference point source for PSF, and then with an object. However, once recorded, the same PSF can be used for different objects unless the object plane is changed. Limited FOV is another restriction, which is set by the angular memory effect, namely the decorrelation of the scattered intensity field. However, because the limited FOV is set on the field angle, and also because of the variable focal length of the scattering lens, we can extend the field size, as desired for a large object, by increasing the object distance and the aperture size. The variable focal length also provides the opportunity of 3-D imaging by displacing either the diffusing media or the detector in the forward or backward direction so as to change the focal length of the system and bring different parts of the object into the focus.

## Electronic supplementary material

Scatter-plate microscope for lensless microscopy with diffraction limited resolution
