# Supplementary material for: Scatter-plate microscope for lensless microscopy with diffraction limited resolution
Source: Sci Rep. 2017 Sep 6;7:10687. doi: 10.1038/s41598-017-10767-3 (PMC5587816; doi:10.1038/s41598-017-10767-3)
Supplement: Supplementary file 1 — Scatter-plate microscope for lensless microscopy with diffraction limited resolution [file 41598_2017_10767_MOESM1_ESM.pdf]

Scatter-plate microscope for lensless microscopy with diffraction limited resolution

Alok Kumar Singh[1] , Giancarlo Pedrini[1], Mitsuo Takeda[1, 2] and Wolfgang Osten[1]

[1]Institut für Technische Optik and Stuttgart Research Center of Photonic Engineering (SCoPE),

University of Stuttgart, Pfaffenwaldring 9, 70569 Stuttgart, Germany

[2]Center for Optical Research and Education (CORE), Utsunomiya University

Yoto 7-1-2, Utsunomiya, Tochigi, 321- 8585, Japan

Experimental Setup

The sketch of the experimental setup for microscopic imaging using a scattering media is shown in Figure 1 of the main article. Here the schematic diagram is shown with more details in supplementary Figure S1. The light from a Nd:YAG laser with $\lambda = 532$ nm was divided into two parts. One part was coupled into a pig-tail fiber (not shown in the figure) and served as an off-axis reference point source. The other part was made to pass through a rotating ground glass (RD), which serves as a spatially incoherent extended source for object illumination. A transmissive object, 1951 USAF test target was placed close to and in front of the beam splitter (BS); the object was made coplanar to the reference point source for imaging. A variable diameter aperture was placed in front of the scattering layer to adjust the speckle size and also the NA of the system. The CCD was mounted on a moving stage and was placed behind the diffuser at an appropriate distance so that the speckles are well sampled on the pixels. The distances between the object and the CCD plane from the scattering media was varied for different magnifications during the experiments. Once the PSF of the system was recorded using the reference point source, the object was placed in the reference plane, off-axis to the reference point, and another intensity distribution was recorded. The object was then reconstructed by cross-correlating these speckle patterns. A similar procedure was adapted to image the biological samples.

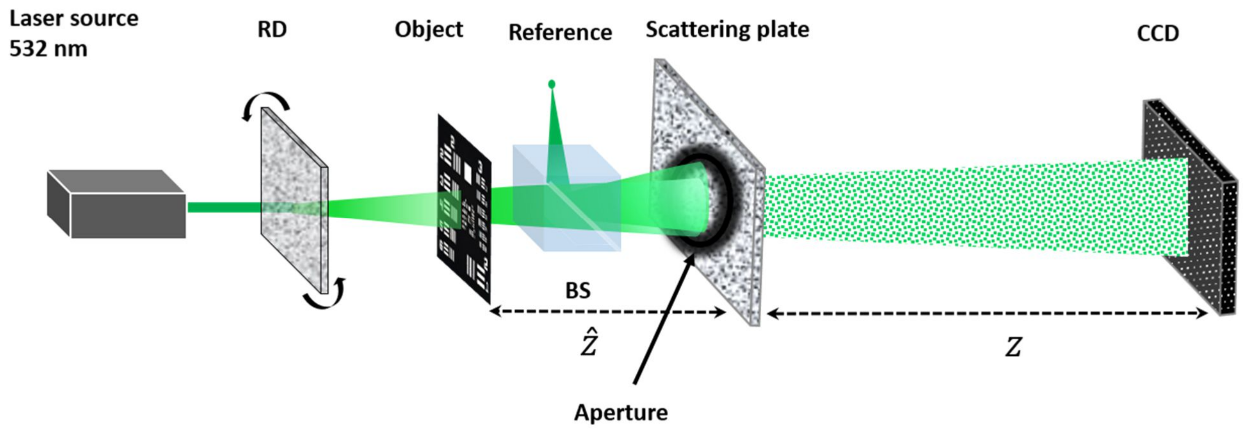

Supplementary Figure S1: Schematic diagram of the 'Scattering plate microscope'. The object and the reference point source are kept coplanar with the help of a beam splitter (BS). A scattering plate is kept very close to the object to image microscopic features of it. The CCD was placed at a distance $z \gg \hat{z}$ to achieve a higher magnification. RD: rotating diffuser and BS: beam splitter.
